# Enhancing glioma immunohistochemical image classification through color deconvolution-aware prior guidance

**DOI:** 10.1371/journal.pone.0324359

**Published:** 2025-09-02

**Authors:** Yiming Gao, Feiyang Niu, Hao Qin, Fang Du, Xiangmei Cao, Lijuan Song

**Affiliations:** 1 College of Information Engineering, Ningxia University, Yinchuan, Ningxia, China; 2 School of Information and Communication, Yinchuan University of Energy, Yinchuan, Ningxia, China; 3 Information Center, General Hospital of Ningxia Medical University, Yinchuan, Ningxia, China; 4 Ningxia Key Laboratory of Artificial Intelligence and Information Security for Channeling Computing Resources from the East to the West, Yinchuan, Ningxia, China; 5 Innovation Center of Ningxia Big Data and Artificial Intelligence Co-construction Collaborative, Yinchuan, Ningxia, China; 6 Department of Pathology, Ningxia Medical University, Yinchuan, Ningxia, China; Yarmouk University, JORDAN

## Abstract

Glioma diagnosis and prognosis heavily rely on immunohistochemistry (IHC), particularly CD34-stained images which highlight tumor vascular endothelial cells. However, traditional image analysis methods struggle with complex staining patterns and subtle morphological variations across glioma subtypes. In this study, we propose a novel Prior-Guided Enhancement Network (PGE-Net) that integrates domain-specific prior knowledge through color deconvolution to enhance feature representation of CD34-positive regions. Unlike existing approaches that treat all pixels equally, our model leverages color abnormality maps to emphasize diagnostically relevant staining patterns, thereby improving both interpretability and classification performance. Experimental evaluation on a curated glioma CD34 dataset demonstrates that PGE-Net achieves notable improvements over ResNet18 baselines, with Precision, Recall, and F1-score increased by 9.17%, 9.35%, and 12.35%, respectively. These results underscore the model’s potential for facilitating more accurate and interpretable IHC image analysis in clinical practice, ultimately supporting more personalized and efficient glioma treatment planning.

## Introduction

Gliomas are among the most common and prognostically poor primary tumors of the central nervous system. Among these, World Health Organization (WHO) grade IV glioblastoma (GBM) has the highest annual incidence, accounting for 54% of all gliomas and 16% of all primary brain tumors.

Recent studies have shown that glioblastoma has surpassed pancreatic and liver cancers to become one of the most challenging tumors to treat [1]. This high mortality rate is primarily attributed to its highly heterogeneous pattern of vascular regeneration, which leads to an almost inevitable recurrence even after treatment [2]. Current treatment strategies—maximal surgical resection, radiochemotherapy, and anti-angiogenic therapy—are often limited by the tumor’s intrinsic heterogeneity, resulting in inadequate accuracy in prognosis assessment. Therefore, developing more reliable diagnostic tools, particularly those capable of identifying precise molecular biomarkers, is crucial for glioma classification, grading, and personalized therapy. Immunohistochemistry, a widely used technique in pathological diagnosis and prognosis evaluation, allows for the visualization of specific proteins in tissue samples through targeted antigen–antibody binding. In routine pathological analysis, hematoxylin is used to stain cell nuclei blue, while diaminobenzidine (DAB) stains target protein regions a brownish color, highlighting areas with positive expression or visually distinct staining [3]. These contrast-enhancing methods assist pathologists in detecting critical disease markers, which directly influence tumor grading and patient survival outcomes.

This study focuses on IHC images obtained using CD34 antibodies to stain glioblastoma tissues. CD34, a membrane-bound protein specific to vascular endothelial cells, is a commonly used marker for studying angiogenesis. It serves as a key biomarker for assessing vascular density and the extent of neovascularization [4]. Research has shown that CD34 is significantly overexpressed in high-grade gliomas [5]. Applying CD34 staining to glioblastoma samples enables the estimation of microvessel density [6] and aids in identifying outlier survival cases [7]. Moreover, the density of CD34-stained microvessels is closely associated with clinical progression and survival outcomes in glioma patients [8]. Therefore, in-depth analysis of image-based CD34 expression not only reflects tumor aggressiveness but also offers valuable molecular insight for precision medicine.

In clinical practice, the interpretation of IHC-stained slides primarily relies on pathologists’ visual assessments and semi-quantitative scoring under a microscope, based on personal experience. However, this traditional approach is often limited by its subjectivity, time-consuming nature, and poor scalability for large-scale image analysis. In recent years, with the growing adoption of Whole Slide Imaging (WSI), conventional glass slides can now be digitized into high-resolution images with wider fields of view, allowing for storage, sharing, and remote analysis [9]. This advancement lays a solid foundation for the automation and standardization of IHC analysis. In WSI-based evaluation, pathologists typically focus on two key aspects: the staining intensity and the proportion of positively stained cells, in order to assess biomarker expression levels. For instance, in the case of CD34, a commonly used semi-quantitative scoring system is applied: staining intensity is scored on a scale from 0 to 3, while the proportion of positive cells is scored from 0 to 4. The sum of these two scores is then used to determine the overall expression level of the biomarker [10].

However, the manual slide analysis approach has two significant limitations: first, when processing WSI images, pathologists are prone to visual fatigue, leading to low scoring efficiency (with an average time of 15–30 minutes per image); second, the scoring results are often inconsistent due to subjective differences among observers, which affects diagnostic stability and the accuracy of treatment decisions [11]. In contrast, recent automated analysis methods have shown remarkable advantages in improving both efficiency and consistency. Unlike manual interpretation, computational methods can perform standardized, batch processing of large-scale IHC images, avoiding the effects of visual fatigue and human biases. For example, Mukundan et al. [12] used hand-designed features and machine learning classifiers to assess HER2 staining in breast cancer tissues, while Zakaria et al. [13] developed threshold-based, domain-specific software tools such as OralImmunoAnalyser. Although these methods are effective in specific scenarios, they are often challenging to generalize for glioma pathology. They also tend to have limitations, such as focusing on narrow regions, lacking interpretability, and difficulty integrating domain-specific pathological knowledge. These limitations are especially prominent in the analysis of glioblastoma CD34-stained images. Compared to other tissue types, such as breast cancer, CD34 staining in glioma tissue often exhibits a higher degree of spatial heterogeneity and irregular staining patterns, making image segmentation and feature extraction more challenging. Most existing algorithms struggle to accurately identify these highly variable structures, leading to unstable results in microvessel density estimation and tumor grading, which in turn affects diagnostic reliability. These technical shortcomings could lead to a series of issues in clinical practice, such as inaccurate WHO grading of gliomas, biased patient prognosis assessments, and even complications in treatment planning and adjustments. Therefore, there is an urgent need to develop an automated analysis method that offers good interpretability, integrates glioma-specific pathological prior knowledge, and robustly handles the complexities of CD34-stained images. This approach would significantly enhance the accuracy and clinical utility of glioma pathological evaluations.

To address the aforementioned challenges, this study proposes an innovative solution. We aim to design an automated analysis approach that specifically tackles the key pain points in manual evaluation of IHC images of gliomas [14]. By developing a prior-guided enhancement model based on color deconvolution awareness, this work enables the automated quantification of staining intensity and area features, offering more objective and accurate analysis results. This not only alleviates the workload of pathologists but also improves diagnostic consistency and efficiency, ultimately supporting the optimization of glioma diagnosis and treatment planning. In our study, CD34 is used as a marker of angiogenesis for IHC staining of GBM tissues. We analyze microvessel density and CD34 expression levels to assess tumor malignancy and patient prognosis. Our main innovation lies in the introduction of a CD prior-guided model, which decomposes the different color channels of stained images and automatically evaluates the staining intensity and area of CD34-positive cells, thereby enhancing the understanding of critical pathological features. In addition, the IHC Perception Module within our model directs the analysis toward diagnostic indicators commonly used by pathologists. This approach not only significantly improves diagnostic accuracy but also reduces errors caused by visual fatigue or inter-observer variability, providing a more reliable tool for assisted diagnosis in pathology.

The main contributions of this study are as follows:

- We propose a CD Prior-Guided Module, which accurately isolates positive regions in IHC images through color deconvolution and extracts salient features—such as staining intensity and area—that are highly aligned with pathological diagnostic indicators. This module establishes structured priors focused on chromatic abnormal regions, enabling the model to attend to key pathological information consistent with the regions of interest typically examined by pathologists. Consequently, it facilitates more accurate identification and quantification of critical features.
- A PGE-Net is proposed for the classification tasks of IHC images. Without the need for constructing annotated datasets, the IHC Perception Module effectively guides the model to focus on regions with abnormal staining patterns, emphasizing the staining intensity and area information in these regions. This method not only improves the classification accuracy of the model but also reduces the reliance on extensive annotated data, enabling targeted feature extraction from IHC images and achieving more precise image classification.

The remainder of this paper is organized as follows: The Related work section reviews the existing literature and highlights current research gaps. The Methods section describes the proposed approach, including the overall model architecture and its core modules. The Experimental results and analysis section presents the experimental setup, dataset, and the obtained findings, together with a discussion of their significance. The Discussion and Conclusions sections interpret the results, address challenges and limitations, and outline directions for future work.

## Related work

For the IHC images used in this study, grading is based on the measurement of the intensity and area of abnormal color regions. Larger abnormal color areas with stronger concentrations are more likely to be classified as higher-grade positive expressions. Therefore, the more precise the extraction of abnormal color regions, the better the final analysis and classification of IHC images. This implies that segmentation is a prerequisite for the classification task. For unsupervised segmentation tasks, prior studies have explored various approaches. Kuok et al. [15] investigated the use of Otsu’s thresholding method to eliminate the background, followed by local adaptive thresholding to refine the segmented foreground. While the local adaptive thresholding technique can enhance segmentation in localized regions, it still struggles to handle image noise and irregular areas, particularly when the transition between the target and background is indistinct, limiting its overall effectiveness.Additionally, some studies employed the CD algorithm to extract irregular positively stained blood vessels in IHC images [16]. Others used the CD algorithm as a preprocessing step to separate dyes in Giemsa-stained images, thereby enhancing the segmentation of white blood cells in the regions of interest [17]. However, in IHC images, cross-channel interference between different staining channels and variations in staining intensity can lead to instability in feature extraction results, preventing the model from effectively attending to critical pathological regions.

In recent years, Xiaoyu Li et al. [18] conducted research on processing pathological images using clustering methods, integrating two stages: supervised clustering and metric-based classification. Mariia Sidulova et al. [19] studied the classification of pathological images using three deep clustering algorithms, demonstrating higher clustering consistency compared to previous WSI clinical annotations. However, for IHC images, clustering methods often rely on global feature extraction, which struggles to effectively handle local staining regions, such as the distribution of specific positive cells. This limitation becomes particularly significant when the staining regions of positive cells are small and unevenly distributed, often leading to a severe decline in classification performance for clustering methods.

For the direct classification tasks of IHC images, Mukundan et al. [20] proposed using handcrafted features as the basis for scoring HER2 IHC images. They analyzed features such as image texture, staining membrane connectivity, staining percentage, and saturation levels using logistic regression and SVM classifiers to evaluate HER2-stained tissue samples.Singh et al. [21] proposed a HER2 immunohistochemical image classification algorithm based on biomarker feature descriptors, which achieved four-class classification by leveraging staining intensity and texture features without relying on complex network architectures. The classification results were highly consistent with pathologists’ assessments. These handcrafted or shallow feature-based methods offer advantages in terms of interpretability, but lack the capability to model deep semantic information within the images. As a result, they show limited accuracy and robustness when dealing with common challenges in IHC images, such as staining ambiguity, structural complexity, and expression heterogeneity.

Recent studies have made significant progress in using threshold-based methods to identify positive regions in IHC images. Marina et al. [12] developed the BreastAnalyser software, which automatically measures the area of DAB brown-stained proteins in immunohistochemistry based on their DAB staining intensity, counts cell nuclei, and classifies staining levels. However, this approach relies on fixed color thresholds to classify complex images, which may lead to limited generalizability under variations in staining intensity, image quality, or experimental conditions, making it challenging to adapt to the diversity of real clinical samples. Zakaria et al. [13] proposed the OralImmunoAnalyser software, which uses color thresholding to compute three levels of staining and count epithelial cells layer by layer. Despite its utility, this approach lacks the capacity to model image context and tissue architecture, rendering it sensitive to artifacts and less effective in dealing with uneven staining or indistinct boundaries. These shortcomings limit its precision in more challenging histopathological analyses.

In recent years, with the rapid development of deep learning in medical diagnosis, CNN have been successfully applied to histological image analysis. For segmentation tasks in IHC images, earlier studies have utilized supervised methods to segment positively stained cells [22–24]. For example, in images where the positively stained cells are nuclei, manual annotations are created for each positively stained nucleus in IHC images to serve as ground truth masks, thereby improving the accuracy of nuclear segmentation tasks. In these studies, the mask data is annotated by pathology experts leveraging their extensive pathological expertise to identify positive regions in IHC images. However, due to the high cost of manual annotation, publicly available IHC datasets with comprehensive annotations are exceedingly scarce. As a result, developing lower-cost and more practical methods has become a promising research direction in the field.

For direct classification tasks on IHC images, researchers have proposed using pretrained networks such as VGG, ResNet, and MobileNet as substitutes for biomarker feature descriptors. These networks extract features from each patch of an IHC image to perform HER2 classification [25,26]. In scenarios where the differences among the four HER2 categories are significant, these models can effectively distinguish the features of the four categories through training on large-scale datasets, achieving efficient classification. Nevertheless, their performance is heavily dependent on the quantity and quality of labeled data, and they exhibit poor generalization in low-data regimes. Furthermore, these approaches often fail to incorporate domain-specific medical knowledge, such as staining mechanisms or tissue architecture, which may lead to “black-box” models with limited interpretability. In addition, some researchers have proposed training separate CNNs for each biomarker—ER, PR, HER2, and Ki67—to classify patches from their respective IHC images and predict the biomarker status individually. The predicted outcomes are then combined to determine the molecular subtype of breast cancer [27]. While this approach leverages biomarker-specific learning, it significantly increases computational cost and deployment complexity. Moreover, training each model independently hinders the discovery of potential inter-marker relationships, thus limiting the overall optimization potential of molecular subtype prediction.

Compared to traditional unsupervised segmentation methods, the proposed approach addresses their limitations in identifying heterogeneous tumor regions by deeply integrating color deconvolution with a depth-aware attention module, offering structured prior support for IHC image analysis. In contrast to clustering-based deep learning models, our method combines color decomposition with an attention mechanism to enable global-level understanding while accurately localizing critical lesion areas in glioma images. This significantly enhances the detection of small, sparsely distributed positive staining regions. Unlike handcrafted or shallow feature-based techniques, the proposed model retains interpretable features informed by pathological knowledge through the integration of color deconvolution and deep attention. This leads to a richer semantic understanding of staining regions. Furthermore, in comparison with threshold-based methods, our approach demonstrates greater robustness under complex backgrounds, allowing for more precise identification of key positive regions and improved adaptability to clinical pathology tasks.

## Methods

To effectively capture the global semantic representation of IHC images, this study proposes a PGE-Net for IHC image classification. The proposed method consists of two main modules: the IHC Perception Module and the CD Prior-Guided Module. The IHC Perception Module utilizes the Feature Importance Network (FIN) [28] to extract features from IHC images. In the following sections, the structure of the PGE-Net is first introduced in detail. The CD prior-guided module then explains the principles of the color deconvolution algorithm used to obtain color abnormality maps. The IHC perception module focuses on the FIN employed in the IHC Perception Module. The detailed procedures for dataset construction, patch generation, and labeling are provided in Dataset and experimental setup.

### Prior-guided enhancement network

**Fig 1:**
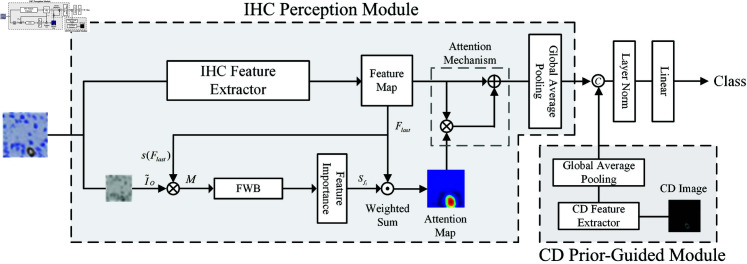
The overall architecture of the Prior-Guided Enhancement Network.

To address the issue of weak feature extraction capability for key metrics, the PGE-Net incorporates color abnormality maps extracted through the color deconvolution algorithm as an external information source, integrating them with the features of IHC images. The overall architecture of the PGE-Net is shown in Fig 1, where the upper gray box represents the IHC Perception Module, and the lower part represents the CD Prior-Guided Module. These two core modules leverage spatial-semantic features and prior knowledge from color decomposition to the fullest extent.

The main purpose of the IHC Perception Module is to process raw IHC images and extract multi-scale spatial and semantic features. The module first uses a backbone network $f_{o}\left(\cdot \right)$ to extract features from the raw IHC image $I_{o}\in R^{H\times W\times 3}$, generating a multi-channel feature map $f_{o}\left(I_{o}\right)\in R^{16\times 16\times c}$, where *c* represents the number of feature channels. This process integrates prior knowledge from contrastive learning models. To further highlight key staining regions, the module employs an attention-based feature weighting mechanism. It converts the original image *I*_*o*_ into a grayscale image and downsamples it to match the spatial resolution of the feature map. The grayscale image is then combined with the normalized feature map to generate an attention mask, which is used to localize stained regions. Subsequently, the feature weighting module enhances the feature representation of important regions, ultimately producing an enhanced feature map to improve classification performance. The primary objective of the CD Prior-Guided Module is to extract prior information related to staining intensity through color decomposition techniques, complementing the spatial-semantic features from the IHC Perception Module. This module processes the input color-decomposed image $I_{p}\in R^{H\times W\times 1}$, which is generated from the raw image using standard color decomposition methods. An independent feature extractor $f_{p}\left(\cdot \right)$ processes *I*_*p*_, generating a feature representation $f_{p}\left(I_{p}\right)\in R^{16\times 16\times c}$,where *c* is consistent with the feature channel dimensions in the IHC Perception Module. The extracted features undergo a global average pooling operation to produce compact prior feature representations of the staining intensity, capturing global staining patterns. These features are then fused with the features from the IHC Perception Module, further enhancing classification performance.

The extracted features contain information about the extent and intensity of positive staining in the immunohistochemical image. In this image, as it only includes the positive staining regions, it partially provides information about the range of positive staining. Additionally, within the positive staining regions, the value of each pixel, i.e., the grayscale intensity, reflects the concentration of positive staining. The higher the grayscale value, the deeper the concentration, thus providing information about the intensity of positive staining.

During the color decomposition process, the resulting data may exhibit a certain degree of sparsity (e.g., dye concentrations in background regions are close to zero). This data sparsity can lead to issues such as feature loss or noise amplification when processed by the model. To address this challenge, we designed a fusion mechanism that combines the decomposed channel information $F_{p}\in R^{1\times 1\times c}$ with the global features of the original RGB image $F_{o}\in R^{1\times 1\times c}$ resulting in a fused representation $F\in R^{2\times c}$. This fusion strategy preserves the biologically relevant features provided by color decomposition while effectively mitigating the negative effects of sparse data, thereby enhancing the model’s robustness. By doing so, the model can better leverage key biomarker information in IHC images while avoiding misclassification caused by background noise or feature sparsity.

The fused features are subsequently passed through a Layer Normalization layer and a Linear layer, ultimately generating the classification results. This process enables the model to integrate positive staining information into multi-class tasks and trains the entire network using a cross-entropy loss function. The design ensures that the network not only captures the overall features of IHC images but also focuses on the range and intensity characteristics of positive staining, improving both the model’s generalization capability and interpretability. This approach is particularly beneficial for non-negative IHC images, where classification relies primarily on the extent and intensity of positive staining. If only raw image features are extracted, the model may fail to concentrate on specific features relevant to classification, leading to insufficient classification criteria

The introduction of the CD Prior-Guided Module provides the model with effective and targeted feature information aligned with the diagnostic criteria used by pathology experts, ensuring the reliability of classification results. To address variations in staining quality of IHC slides, the CD Prior-Guided Module extracts normalized features from the staining channels, significantly reducing the impact of staining intensity and tonal variations on model performance. Meanwhile, the IHC Perception Module employs a dynamic attention mechanism to automatically identify and focus on high-confidence positive staining regions, minimizing interference from noise and staining inconsistencies. This design not only enhances the model’s robustness under varying staining quality but also further improves classification performance, meeting the practical requirements of pathological analysis.

### The CD prior-guided module

To address the issue where the model cannot effectively extract specific features from the focused regions, we introduced the color deconvolution algorithm. This approach enhances the model’s deep semantic analysis capability of IHC images by incorporating external knowledge. The specific features refer to the intensity and area characteristics of the color anomaly regions, while the external knowledge is represented by the color anomaly maps obtained from the positive staining regions of the IHC images through the color deconvolution algorithm. To efficiently utilize this external knowledge, the CD Prior-Guided Module’s feature extractor processes the color anomaly maps to extract the intensity and area characteristics of these regions. This process is intended to align with the key metrics that pathologists typically focus on during diagnosis, thereby improving the model’s interpretability and accuracy in identifying relevant pathological features.

**Fig 2:**
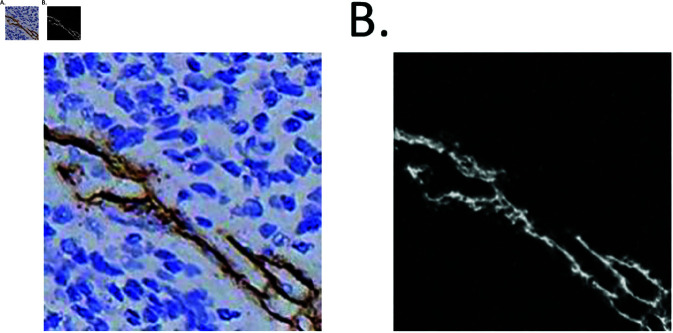
Comparison of IHC and color anomaly image. (A) IHC image showing tissue staining patterns.(B) Color anomaly image highlighting deviations in chromatic features.

For pathological images, there are various staining methods used, but generally, they involve the use of two or three different dyes for staining [29,30]. Taking advantage of the relatively limited number of color categories in such images, Ruifrok and colleagues [31] proposed the color deconvolution algorithm, which is better suited for the separation and quantitative analysis of staining in these types of pathological images. Immunohistochemistry staining is an essential technique in modern pathology diagnostics and plays a crucial role in the diagnosis of various diseases. One of the most commonly used staining methods in IHC is DAB staining [32]. DAB staining results in the appearance of a light yellow, brown-yellow, or brown color at the antigen-binding sites where it reacts with the primary antibody. The intensity of this color reflects the quantity of the antigen present, serving as the basis for the qualitative, spatial, and quantitative analysis of IHC samples. Additionally, hematoxylin is used to counterstain the cell nuclei in tissue sections, staining them blue. Through a series of processing steps, immunohistochemistry images containing colors from both staining agents are obtained. The purpose of applying the Color Deconvolution algorithm in this study is to isolate the DAB-stained regions, which correspond to the positive areas in the immunohistochemistry images, as illustrated in Fig 2. Fig 2(a) represents the original IHC image, while Fig 2(b) shows the color deconvolution result, where the DAB-stained channel in the IHC image has been extracted and reconstructed, resulting in the color abnormality image.

Here is the explanation of the Color Deconvolution algorithm’s principle: According to the Lambert-Beer Law, it is known that the intensity of transmitted light depends on the concentration of the dye in a nonlinear manner. Consequently, the grayscale values in each channel also exhibit nonlinear dependencies on the dye concentration. As a result, the grayscale values of an image, specifically in the R, G, and B color channels, cannot be directly utilized for the separation and quantitative analysis of staining in immunohistochemistry images. The Lambert-Beer Law is represented by Eq (1):

$$I_{c}=I_{o,c}\exp\left(-Ac_{c}\right)$$ (1)

In this formula, *I*_*o*,*c*_ represents the intensity of incident light, *I*_*c*_ represents the intensity of transmitted light, *A* refers to the amount of dye, is the specific absorption factor of light in a particular channel, and the subscript C indicates a specific color channel.

However, the optical density values (OD), also known as absorbance, directly correlate with the quantity of the dye and follow the Lambert-Beer Law. These OD values can be used directly for quantifying immunohistochemistry images. The relationship between optical density and substance content adheres to the following principle: at a specific wavelength, when the target color is darker, less light passes through, resulting in a higher optical density value. Thus, optical density can measure the content of a particular component within tissues or cells. As an example, in immunohistochemical reaction results, for quantitative analysis of the stained antibody quantity, the actual process involves the quantitative measurement of antibody staining after mathematical calculations on image data.

The optical density of each channel is defined as shown in Eq (2):

$$OD_{c}=-\log_{10}\left(I_{c}/I_{o,c}\right)=A*c_{c}$$ (2)

*OD*_*c*_ refers to the optical density value corresponding to each color channel (such as the red, green, or blue channel). Here, *I*_*o*,*c*_ denotes the incident light intensity, and A* represents the absorption coefficient. From Eq (2), it can be observed that the OD value for each channel exhibits a linear relationship with the dye concentration. Therefore, it is possible to separate the staining components of samples in the OD space. The characteristics of each dye are the specific optical densities for each channel in the three RGB channels, and they can be represented by a 3 × 1 OD vector, which is used to describe the conversion from RGB color space to the OD space. By measuring the relative absorbance of red, green, and blue colors on glass slides stained with a single dye, one can determine the OD values for each channel (R, G, B). For example, if a sample is stained only with hematoxylin, the OD values for the R, G, and B channels correspond to 0.18, 0.20, and 0.08, respectively.

However, in pathological staining, multiple dyes are often used, resulting in stained images with several coexisting colors. When three dyes are used for staining, the color matrix X of staining with three dyes is represented as shown in Eq (3):

$$X=[\begin{array}{ccc} p_{11} & p_{12} & p_{13}\\ p_{21} & p_{22} & p_{23}\\ p_{31} & p_{32} & p_{33} \end{array}]$$ (3)

In matrix X, each row corresponds to a specific dye, and each column corresponds to the optical densities detected for each dye in the R, G, and B channels.

For instance, when three dyes, namely hematoxylin, eosin, and DAB, are used for staining, an example of the OD matrix for this combination is shown in Eq (4):

$$M=[\begin{array}{ccc} 0.18 & 0.20 & 0.08\\ 0.01 & 0.13 & 0.01\\ 0.10 & 0.21 & 0.29 \end{array}]$$ (4)

The matrix values referenced here are based on the study by Ruifrok et al. In order to separate the three stains in pathological images, it is necessary to standardize the OD matrix, achieving the correct balance of absorption factors for each individual stain. The standardization process involves orthogonal and normal transformation of the RGB information to obtain independent information contributions for each dye. The normalization process involves dividing each OD vector by its total length, as shown in Eqs (5), (6), and (7):

$${\hat{p}}_{11}=\frac{p_{11}}{\sqrt{p_{11}^{2}+p_{12}^{2}+p_{13}^{2}}}$$ (5)

$${\hat{p}}_{21}=\frac{p_{21}}{\sqrt{p_{21}^{2}+p_{22}^{2}+p_{23}^{2}}}$$ (6)

$${\hat{p}}_{31}=\frac{p_{31}}{\sqrt{p_{31}^{2}+p_{32}^{2}+p_{33}^{2}}}$$ (7)

This results in the standardized OD matrix $\hat{X}$, as shown in Eq (8):

$$\hat{X}=[\begin{array}{ccc} {\hat{p}}_{11} & {\hat{p}}_{12} & {\hat{p}}_{13}\\ {\hat{p}}_{21} & {\hat{p}}_{22} & {\hat{p}}_{23}\\ {\hat{p}}_{31} & {\hat{p}}_{32} & {\hat{p}}_{33} \end{array}]$$ (8)

The standardized OD matrix $\hat{M}$ for hematoxylin, eosin, and DAB is shown in Eq (9):

$$\hat{M}=[\begin{array}{ccc} 0.65 & 0.70 & 0.29\\ 0.07 & 0.99 & 0.11\\ 0.27 & 0.57 & 0.78 \end{array}]$$ (9)

Assuming C is a 3×1 vector representing the contribution of hematoxylin, eosin, and DAB stains on each pixel. In the OD space, the OD vector of this pixel is $y=C\hat{M}$. Then, $C={\hat{M}}^{-1}[y]$, meaning that the OD values for each pixel in the image are multiplied by ${\hat{M}}^{-1}$. The inverse matrix of the OD matrix, denoted as the color deconvolution matrix D, is $D={\hat{M}}^{-1}$. For the OD matrix $\hat{M}$ representing hematoxylin, eosin, and DAB, the color deconvolution matrix D is shown in Eq (10):

$$D=[\begin{array}{ccc} 1.88 & -0.07 & -0.60\\ -1.02 & 1.13 & -0.48\\ -0.55 & -0.13 & 1.57 \end{array}]$$ (10)

Finally, you obtain *C* = *D*[*y*]. When you want to extract a specific stain from the pathological image, you can access the contribution value of that stain in the C vector, allowing for quantification. In this context, your original OD matrix O, as the image only contains hematoxylin and DAB stains, is shown in Eq (11):

$$O=[\begin{array}{ccc} 0.18 & 0.20 & 0.08\\ 0.10 & 0.21 & 0.29 \end{array}]$$ (11)

### The IHC perception module

**Fig 3:**
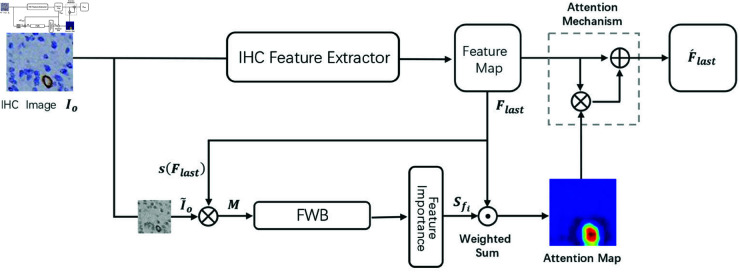
Overall IHC Perception Module architecture.

In the IHC Perception Module, this study employs the FIN for image feature extraction. The attention mechanism within FIN leverages generated attention maps to enable the model to focus on the positive staining regions of IHC images, which are the areas of interest for pathologists. These attention maps provide higher visual interpretability, as they are generated by the Feature Weighting Module (FWB), which learns the importance of various features in IHC images. In the CD Prior-Guided Module, ResNet18 is utilized for image feature extraction. The FIN enhances ResNet18 by introducing channel attention, which guides the model to focus on regions with color abnormalities in IHC images, thereby further optimizing the model’s decision-making capabilities. The overall architecture of the IHC Perception Module is illustrated in Fig 3.

The input image *I*_*o*_ first goes through the IHC feature extraction network ResNet18 to obtain multi-channel feature maps *F*_*last*_, which serve as the basis for FIN to provide different channel feature importance scores. Next, *I*_*o*_ is transformed from the RGB color space to a single grayscale space and downsampled to the same size as the single-channel feature map, resulting in ${\tilde{I}}_{o}$, calculated as shown in Eq (12):

$${\tilde{I}}_{o}=Down(rgb2gray(I_{o}))$$ (12)

Here, S(·) represents mapping each element of each feature map to the range [0, 1], which is normalization applied to *F*_*last*_. $F_{last}^{c}$, as a multi-channel feature map, is obtained from the last convolutional layer of the feature extraction network, and normalization is applied to all c channels of the feature maps. The mask image *M*_*c*_ is then calculated by multiplying the downsampled grayscale image ${\tilde{I}}_{o}$ and the normalized feature map $s(F_{last}^{c})$, as shown in Eq (13):

$$M_{c}={\tilde{I}}_{o}⊗s(F_{last}^{c})$$ (13)

Once a set of mask images $\left\{M_{1},M_{2},\ldots ,M_{c}\right\}$ is obtained, they are concatenated into the final mask image M, which is then input into the FWB for forward propagation, as shown in Eq (14):

S=FWB(M) (14)

Here, S is a vector of feature importance scores, $S\in R^{c}$, where each score ${\text{S}}^{\text{c}}$ represents the weight assigned to the importance of the corresponding feature map.

After obtaining the importance scores for each feature map, they can be weighted and combined separately with their corresponding feature maps to generate the final class activation maps for FIN, denoted as *L*_*LFI*−*CAM*_, as shown in Eq (15):

$$L_{LFI-CAM}=\text{Re}LU\left(\sum_{c=1}^{N}S^{c}F_{last}^{c}\right)$$ (15)

Here, ReLU is applied to the linear combination of feature maps to remove features with negative influences. Before obtaining reliable output results, the attention mechanism is mainly used to assist the classifier in making more accurate decisions. In this mechanism, $F_{last}^{c}$ represents the feature maps of the last convolutional layer, and ${\hat{F}}_{last}^{c}$ is the output of the attention mechanism, as shown in Eq (16):

$${\hat{F}}_{last}^{c}=(1+L_{LFI-CAM})⊗F_{last}^{c}$$ (16)

The class activation map *L*_*LFI*−*CAM*_ in the attention mechanism of FIN serves as the attention map, highlighting the high-value regions in the feature maps to guide the model’s focus on important areas. These important areas refer to regions that are more consistent with the attributes of the image, and the image attributes are the image category labels defined before training. When these attributes align more closely with the interpretation of image information, the model is more likely to output the results humans expect. Therefore, FIN not only explains how the model understands input images but also makes model decisions more controllable and accurate.

### Algorithm process

In this study, we designed a glioma IHC image classification algorithm enhanced by CD-Aware Prior Guidance. This algorithm aims to address the challenge of the model’s inability to extract specific features from focused regions, particularly in the accurate assessment of staining intensity and the area of positive cells. By integrating deep learning with channel attention mechanisms, our algorithm enables automated and precise analysis and classification of IHC images, with a particular emphasis on regions with color abnormalities. This approach holds significant potential for improving the accuracy of glioma diagnosis and prognosis evaluation.

We designed the glioma IHC image classification algorithm proposed in this study, with the algorithm framework outlined in Algorithm 1. Here, ϕ(·) represents adaptive average pooling, Concat(·) indicates feature concatenation, LN(·) represents the LayerNorm layer, and FC(·) denotes the fully connected layer.

**Algorithm 1. Glioma IHC image classification algorithm enhanced by CD-Aware Prior Guidance.**

**Require:** Immunohistochemical image *I*_*o*_, Color Aberration

Image *I*_*p*_

**Ensure:** Immunohistochemical image class L

1: Randomly initialize network weights

2: ${I^{\prime }}_{o}=f_{o}\left(I_{o}\right)$

3: **while** c<C **do**

4:   $M_{c}=Down(rgb2gray(I_{o})⊗s(I^{\prime }{\;\;}_{o}^{c}))$

5: **end while**

6: M← Concatenate the mask images $\left\{M_{1},M_{2},\ldots ,M_{c}\right\}$

7: $S=FWB(M),S\in R^{c}$

8: **while** c<C **do**

9:   $w_{c}=S^{c}$

10:   $X_{o}^{c}=(1+\text{ Re}LU(\sum_{c=1}^{N}w_{c}I^{\prime }{\;\;}_{o}^{c}))⊗I^{\prime }{\;\;}_{o}^{c}$

11: **end while**

12: $X_{o}\leftarrow$ Concatenate the feature maps $\left\{X_{o}^{1},X_{o}^{2},\ldots ,X_{o}^{c}\right\}$

13: $F_{o}=\phi (X_{o})$

14: $F_{p}=\phi (f_{p}(I_{p}))$

15: $L=FC(LN(Concat(F_{o},F_{p})))$

16: **return** L

## Experimental results and analysis

In this section, we provide a detailed description of the dataset and experimental setup for IHC image classification in this study. In the Dataset and experimental setup section, we describe the dataset and experimental settings for IHC image classification. In the Experimental results and analysis section, we compare our proposed PGE-Net with various baseline models and quantitatively evaluate its performance in IHC image classification.

### Dataset and experimental setup

This study utilized two datasets: the publicly available DeepLIIF dataset [33] and the CD34 glioma IHC image dataset provided by the Affiliated Hospital of Ningxia Medical University (hereafter referred to as the CD34 dataset). The primary experiments were conducted on the CD34 dataset. Although the data were sourced from the Affiliated Hospital of Ningxia Medical University, this regional medical center serves a wide catchment area, treating glioma patients from diverse regions. The patient cohort spans different ages, genders, tumor grades, and anatomical locations, ensuring diversity in geographic distribution and population characteristics. To validate the generalization capability of the proposed method, additional experiments were conducted on the public DeepLIIF dataset. The IHC slides in the DeepLIIF dataset come from various sources and exhibit natural variations in staining intensity and quality. Consistent results on this dataset demonstrate that the model effectively mitigates the impact of staining differences on classification performance through color decomposition and attention mechanisms.

The DeepLIIF dataset consists of 575 training images, 91 validation images, and 598 test images, each with a resolution of 512 × 512 pixels. These images are 40x magnified immunohistochemical images of bladder cancer and non-small cell lung cancer slides. The trained DeepLIIF model can generate corresponding Hematoxylin, mpIF DAPI, mpIF Lap2, mpIF Ki67 channel images, and multiclass cell segmentation masks from the input immunohistochemical images. Additionally, Ghahremani et al. provided a dataset in the same format as the DeepLIIF dataset, named BCDataset [34]. This dataset includes 385 training images and 66 validation images, each with a resolution of 512 × 512 pixels, and consists of Ki67-stained breast cancer slide images.

Due to the limited size of the BCDataset, it is insufficient for standalone model training and testing. However, given the similarity in structure and Ki67 staining between the BCDataset and the DeepLIIF dataset, these two datasets were combined and collectively referred to as the DeepLIIF dataset in this study. After organizing the DeepLIIF dataset, we obtained 1,715 negative images and 1,598 non-negative images, with a training-to-validation split ratio of 6:4.

The CD34 dataset was collected by our team at the Affiliated Hospital of Ningxia Medical University, constructed based on real clinical cases. All images were derived from immunohistochemically stained slides in the pathology department and were manually evaluated and scored by experienced pathologists. Strict quality control measures were implemented to ensure the accuracy and consistency of annotations. The dataset includes 40 WSIs of immunohistochemical staining. Due to the high computational complexity of directly analyzing large-scale WSI images, they were divided into smaller patch images for analysis. In this study, the commonly used patch size of 256 × 256 was adopted for segmentation. A background screening step was also implemented to remove blank or non-informative patches, thereby reducing computational load and enhancing the quality of data for model training. To improve the generalizability of the model and alleviate training bias stemming from class imbalance, we employed a range of data augmentation techniques during the training phase, such as rotation, cropping, and color jittering.

In the processed CD34 dataset, the patches were categorized into two main classes: negative and non-negative. The non-negative patches were further divided based on CD34 expression levels into three subcategories: low expression (weak positive), medium expression (positive), and high expression (strong positive). Under the guidance of pathologists’ scoring, the dataset was finalized with 2,437 negative images, 606 weak positive images, 773 positive images, and 742 strong positive images. The dataset was randomly split into training and validation sets at a ratio of 7:3. The sample distribution of each category in the training and validation sets is shown in Table 1:

**Table 1:** Categories and Quantities of the CD34.

Image Category	Number in Training Set	Number in Validation Set
negativ	1,705	732
weak positive	424	182
positive	541	232
strong positive	519	223

In the experiments, we utilized the SGD optimizer with a total of 300 epochs for parameter updates. The initial learning rate was set to 1 × 10^−5^, with a learning rate adjustment multiplier defaulted to 0.1. Weight decay (L2 regularization) was set to a default value of 1e-4. The batch size for training was set to 256, and for testing, it was set to 200. During training, both the training set batch size and validation set batch size were set to 32. The learning rate was reduced by a factor of 10 at 50% and 75% of the total training epochs. CrossEntropyLoss was employed as the loss function for this experiment. All experiments were conducted in the same environment, and the experimental environment configuration is presented in Table 2.

**Table 2:** Experimental environment configuration.

Environment/Language	Version/Model
Python	3.7.0
Cuda	10.2
Pytorch	1.12.0
GPU	NVIDIA TITAN RTX 24G*2
CPU	AMD Ryzen 9 3950X 16-Core Processor
Ubuntu	20.04.4 LTS

### Experimental results and analysis

To evaluate the effectiveness of incorporating prior knowledge, this study compares the performance of the PGE-Net and the baseline model, ResNet18 [35], on two datasets. The core distinction of the PGE-Net lies in the introduction of the CD Prior-Guided Module. This module extracts features from the color anomaly map corresponding to IHC images, enabling the network to focus more on regions with abnormal features. These feature regions serve as additional prior inputs, which are passed to the classification model, allowing it to acquire a set of prior information that aids classification beyond the basic data input. Furthermore, the IHC Perception Module extracts features from IHC images, and with the support of the attention mechanism, the model can more accurately focus on positive staining regions.

In this study, Precision, Recall, and F1-score are employed as the primary evaluation metrics. These metrics are widely used in medical image analysis and are particularly suitable for tasks involving class imbalance. Precision helps reduce false positives and prevents overdiagnosis; Recall mitigates the risk of missed diagnoses; and the F1-score provides a balanced assessment of the model’s stability and reliability in identifying positive regions. Although other clinically relevant metrics were not adopted, the current evaluation framework possesses a certain degree of clinical interpretability and can indirectly reflect the model’s practical value in real-world diagnostic workflows.

On the DeepLIIF and CD34 datasets, the performance of the PGE-Net and the baseline model was compared, with the specific results shown in Tables 3 and 4. The experiments evaluated the impact of each component of the model on its performance. The baseline model uses ResNet18 solely as a feature extractor. Additionally, comparisons were made regarding the inclusion of the CD Prior-Guided Module in the model, as well as the performance of different feature extractors within the IHC Perception Module.

**Table 3:** The comparison results between the PGE-Net and the baseline model on the DeepLIIF dataset.

Method	Params	Precision	Recall	F1-score
ResNet18 (BaseLine)	11.18M	0.8181	0.8009	0.7971
FIN	20.62M	0.8471	0.8379	0.8362
FIN+MLP-Mixer [36]	31.27	0.9246	0.9238	0.9237
PGE-Net	**31.79M**	**0.9379**	**0.9367**	**0.9367**

**Table 4:** The comparison results between the PGE-Net and the baseline model on the CD34 dataset.

Method	Params	Precision	Recall	F1-score
ResNet18 (BaseLine)	11.18M	0.8234	0.8225	0.7906
ResNet18 + ResNet18	22.35M	0.9012	0.9021	0.8987
FIN	20.62M	0.8561	0.8561	0.8419
FIN+MLP-Mixer	31.27M	0.9015	0.9007	0.8967
PGE-Net	**31.79M**	**0.9151**	**0.9160**	**0.9141**

To validate the significance of the improvements in performance metrics such as Precision, Recall, and F1-score, we conducted statistical significance tests on the experimental results for each group. In the experiments, each test group was subjected to multiple independent trials (n = 5) to ensure the stability of the results. As shown in Table 3, compared to the baseline model using only ResNet18, the PGE-Net achieved significant improvements in all metrics, with Precision, Recall, and F1-score increasing by 14.64%, 16.94%, and 17.05%, respectively. Furthermore, compared to methods based on FIN for extracting IHC features, the proposed model also demonstrated superior performance across all metrics. These findings indicate that the integration of the CD Prior-Guided Module and the IHC Perception Module effectively enhances the classification model’s ability to recognize positive staining regions by leveraging prior knowledge and domain-specific features.

As shown in Table 4, on the CD34 dataset, the performance of the baseline model, the dual ResNet18 model, and the model using FIN for IHC feature extraction was slightly lower than that of the PGE-Net. Compared to the dual ResNet18 model, the PGE-Net achieved improvements of 1.54%, 1.54%, and 1.71% in Precision, Recall, and F1-score, respectively. Notably, regardless of whether FIN or ResNet18 was used for the IHC Perception Module, incorporating the CD Prior-Guided Module consistently resulted in significantly better performance across all metrics compared to models without the CD Prior-Guided Module.

In the experiments conducted on the CD34 dataset, the proposed PGE-Net utilized FIN for extracting IHC image features and ResNet18 for extracting features of color anomaly maps. The experimental results demonstrate that this model achieved the best performance across all metrics, with Precision, Recall, and F1-score improving by 9.17%, 9.35%, and 12.35%, respectively, compared to the baseline. It is worth noting that regardless of whether the IHC Perception Module adopts FIN or ResNet18, the integration of the CD Prior-Guided Module consistently leads to significant improvements across all performance metrics compared to configurations without the prior-guided module. When the FIN module incorporates the CD Prior-Guided Module—regardless of whether the feature extractor within the CD module is MLP-Mixer or ResNet18—the model performance is markedly enhanced. This demonstrates that the color anomaly maps captured by the CD Prior-Guided Module effectively assist FIN in the classification of IHC images. In particular, the performance improvement is especially prominent on the DeepLIIF dataset. This is because, in our construction of the DeepLIIF dataset, only two attribute categories are defined: negative and non-negative. As such, the positive information provided by the CD Prior-Guided Module helps to better amplify the feature distinctions between these two categories, thereby reducing the model’s discriminative burden and facilitating more accurate differentiation between image types. Moreover, across both datasets, the ResNet18-based feature extractor within the CD Prior-Guided Module slightly outperforms the MLP-Mixer-based extractor. In our implementation, the depth of the Mixer Layer in the MLP-Mixer model is set to 4, allowing the transformed tokens to pass through four Mixer Layers. This configuration ensures that the resulting model has a parameter count comparable to ResNet18, enabling a fairer comparison between the two architectures.

**Fig 4:**
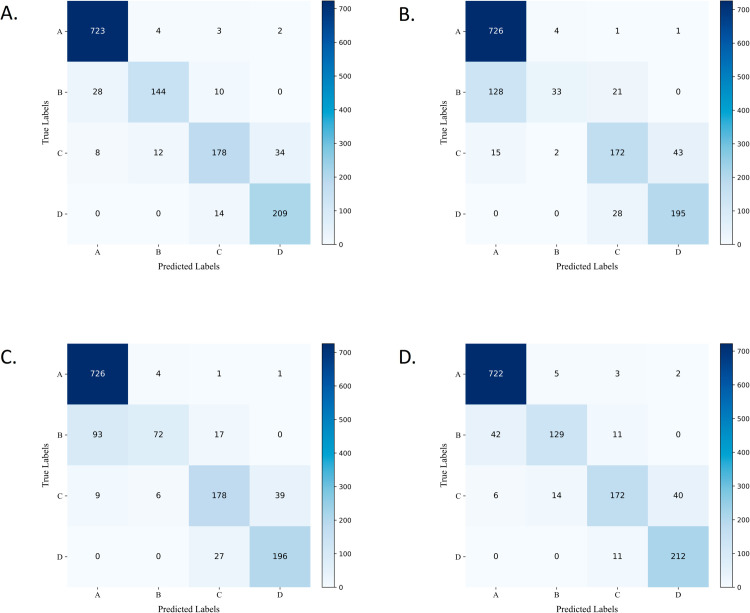
Confusion matrix of different method. (A) PGE-Net (B) BaseLine (C) FIN (D) BaseLine + ResNet18.

Using the trained model to classify the validation set, images were categorized into four classes: negative (A), weakly positive (B), positive (C), and strongly positive (D). The confusion matrix of the classification results is shown in Fig 4. As illustrated, the PGE-Net demonstrates a significant advantage in identifying weakly positive patches. The confusion matrix provides a tabular representation of the relationship between the true class labels and the predicted results of a classification model. It summarizes the number of correct and incorrect predictions for each class. In this matrix, the total number of entries in each column represents the number of instances predicted as that class, while the total number in each row indicates the actual number of instances belonging to that class. A classification model demonstrates better performance when the values of True Positives (TP) and True Negatives (TN) are higher, and the values of False Positives (FP) and False Negatives (FN) are lower.

**Fig 5:**
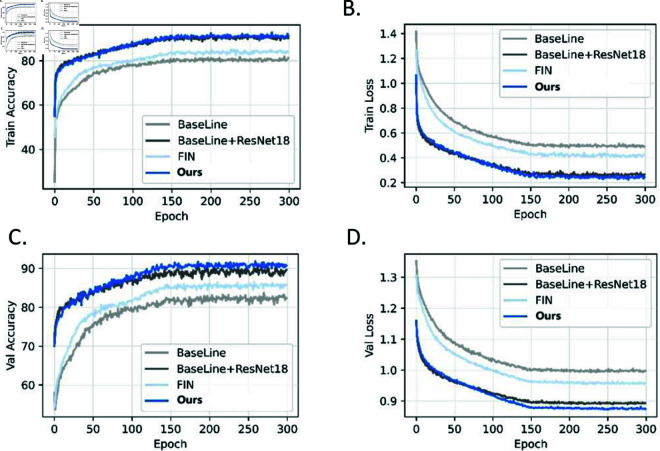
Accuracy and loss curve of different methods. (A) Training set accuracy curve. (B) Training set loss value curve. (C) Verification set accuracy curve (D) Verification set loss value curve.

**Fig 6:**
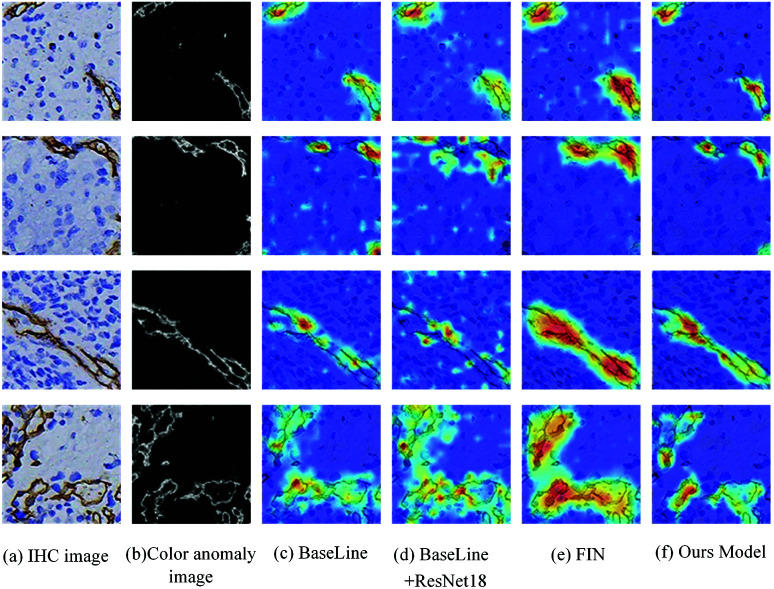
Visual interpretation results of different methods on IHC images. (a) IHC Image: Original immunohistochemical image showing DAB-stained regions and hematoxylin-stained nuclei. (b) Color Anomaly Image: Highlights significant staining intensity using the model’s color decomposition module. (c) Baseline: Heatmap from the basic model, with limited feature clarity and potential signal loss. (d) Baseline + ResNet18: Improved heatmap with ResNet18, offering better accuracy but some noise and unclear boundaries. (e) FIN: A heatmap generated using an enhanced feature extraction network. The key regions are more prominent, but background noise may still affect some results. (f) Ours Model: Clear and precise heatmap, effectively suppressing noise and accurately localizing key areas.

Furthermore, Fig 5 illustrates the accuracy and loss variations across each epoch for different methods on the training and validation sets of the CD34 dataset. From these trends, it is evident that the PGE-Net achieves faster training convergence and demonstrates more stable performance on the validation set. Fig 6 presents the class activation map visualizations for different methods on the CD34 dataset. The visual analysis further validates that the PGE-Net focuses more effectively on key feature regions, thereby improving classification performance and significantly reducing misclassification. This indicates that by integrating prior information on spatial features and staining intensity, the model achieves substantial improvements in Precision, Recall, and F1-score for the classification task.

As shown in Fig 6, on the CD34 dataset, the FIN model demonstrates more accurate localization of color anomaly regions in IHC images compared to the ResNet18 model and the baseline + ResNet18 model. Accurate localization is essential to ensure the model’s reliability and interpretability in image prediction tasks. Furthermore, incorporating the CD Prior-Guided Module into the FIN model does not compromise its precise localization of color anomaly regions. This indicates that the FIN model and the CD Prior-Guided Module function independently without interfering with each other. At the feature fusion stage, the information from both components is aggregated, enabling the model to maintain a high level of accuracy in localizing color anomaly regions while simultaneously considering the intensity and area characteristics of these regions. In summary, the comparison results demonstrate that the prior-guided enhanced network achieves the best performance.

## Discussion

The proposed Prior-Guided Enhanced Network not only significantly improves the classification performance of glioma IHC images but also effectively integrates domain-specific prior knowledge into the deep learning framework. By leveraging the color deconvolution algorithm to extract and focus on critical pathological regions, this study successfully bridges the gap between traditional pathological practices and modern computational methods. This integration offers dual advantages: enhancing the model’s interpretability while ensuring its clinical relevance. Additionally, the significant improvements in precision, recall, and F1-score further highlight the model’s robustness and reliability, providing strong support for glioma diagnosis and prognostic evaluation. Admittedly, we also recognize that the proposed model still has certain limitations. For instance, when CD34 staining is insufficiently dense or when background noise is enhanced, the model may encounter challenges such as inaccurate localization or uncertain classification. Moreover, different types of gliomas (e.g., low-grade vs. high-grade) exhibit significant differences in both pathological morphology and immunohistochemical expression, which places higher demands on the model’s generalization capability. Future work will focus on developing enhanced mechanisms to address issues related to inconsistent staining intensity and tumor heterogeneity, aiming to improve the model’s robustness in complex pathological scenarios.

In practical clinical diagnosis, the staining quality of IHC slides may vary due to experimental conditions, posing challenges to the stability of classification models [37]. The proposed model offers normalized staining feature representations and incorporates attention mechanisms to dynamically focus on high-confidence regions, effectively mitigating the impact of staining variations on classification performance. This design improves the model’s adaptability, enabling it to better handle the diversity of staining conditions encountered in real-world applications.

Furthermore, to enhance the interpretability of the model, we designed a visualization mechanism based on color anomaly mapping. These color anomaly maps intuitively highlight the regions of interest in diseased tissues, providing clear support for the model’s predictions. For example, in the assessment of cancerous tissues, the anomaly maps effectively mark areas that may exhibit cellular abnormalities, helping pathologists focus on regions requiring deeper analysis. This not only shortens diagnostic time but also improves diagnostic accuracy. Additionally, these maps assist pathologists in identifying tumor boundaries, playing a critical role, particularly in distinguishing benign from malignant cases. This visualization approach adds transparency to the model’s predictions, making it more suitable for clinical decision-making scenarios. However, there is currently a lack of systematic user studies or feedback from pathologists to quantitatively assess the applicability of this method in real-world workflows [38]. To further validate the clinical utility of the model, future work will involve user experience studies with participation from pathology experts. The model will be integrated into a semi-automated diagnostic assistance tool, and usage data and feedback will be collected through simulated diagnostic tasks. This approach will enable a more objective evaluation of the method’s potential to improve diagnostic efficiency and accuracy in practical settings.

Although the proposed model demonstrated promising performance in experimental settings, it has yet to undergo real-time or semi-automated evaluation in actual clinical practice. To facilitate clinical translation, several steps need to be taken: integrating the model into clinical workflows for real-time testing, developing user-friendly interfaces, conducting multi-center validations to ensure robustness across diverse datasets, and providing relevant training for clinical staff. Furthermore, regulatory approval and ethical considerations must be addressed to ensure compliance and safeguard patient data.

## Conclusions

This paper proposes a Prior-Guided Enhancement Network to improve the classification performance of glioma IHC images. The model consists of two core modules: the IHC Perception Module and the CD Prior-Guided Module. The IHC Perception Module leverages an attention mechanism to focus on high-staining regions, extracting spatial-semantic features and effectively capturing deep characteristics within IHC images. Simultaneously, the CD Prior-Guided Module extracts staining intensity-related prior features from color-decomposed images. These prior features serve as additional inputs, working alongside original image features to participate in the classification process, effectively enhancing the model’s focus on positive staining regions. By integrating the features from both modules, the model captures prior knowledge and domain-specific features within the images, providing richer feature representations for classification tasks. This significantly improves model performance and aligns the decision-making process more closely with the analytical logic of pathologists.

Compared to conventional models, our proposed approach introduces color deconvolution priors. Traditional models process raw images without explicitly leveraging prior knowledge of stain-background separation. In contrast, we specifically designed a CD-aware prior-guided module, which extracts prior information such as staining intensity and coverage to provide meaningful complementary features for the classification task. Additionally, in the IHC-aware module, we employ an attention mechanism to generate class activation maps that focus on stained regions, significantly enhancing the model’s ability to attend to critical areas.

The PGE-Net utilizes color decomposition techniques to generate color anomaly maps, enabling the network to focus on tumor-related staining abnormality regions in IHC images. Experimental results show that the PGE-Net improves Precision, Recall, and F1-score by 9.17%, 9.35%, and 12.35%, respectively. These experiments demonstrate the effectiveness and reliability of the proposed method compared to the baseline model, providing a more cost-effective and efficient solution for automated quantitative analysis of IHC images.

Future work will advance the model along multiple dimensions to enhance its generalization capability, interpretability, and clinical applicability. First, we plan to extend training and validation of the model to other common biomarkers, such as PD-L1, Ki-67, and HER2, exploring its potential in a broader range of tumor types. This will improve the model’s generalization across various tissue types and further expand its applicability in real-world clinical settings.

At the architectural level, we aim to optimize the network design to better accommodate the complexity of different staining markers and tissue morphologies. To improve interpretability, we will develop dedicated explanation modules tailored for pathologists. For example, by integrating attention heatmaps, feature importance ranking, and case-level visual summaries, we aim to provide clear and actionable decision support to clinical users, thereby enhancing their trust and willingness to adopt the system.

To meet the demands of complex pathological tasks, we will also develop a unified framework capable of handling multiple tasks, including classification, segmentation, and IHC scoring. This multi-task learning strategy is expected to improve overall performance and better support diverse diagnostic scenarios in routine workflows. Given the prevalence of staining artifacts, tissue fragmentation, and preparation errors in real-world IHC samples, we plan to incorporate artifact-aware augmentation strategies, together with adaptive normalization and robust training mechanisms, to improve the model’s tolerance to inconsistent staining conditions and ensure stable performance in challenging environments.

Furthermore, we aim to develop the model into an efficient, real-time interactive assistance system that can be seamlessly integrated into mainstream digital pathology platforms, such as WSI systems. This deployment paradigm will not only assist pathologists in expediting diagnostic workflows but also significantly improve efficiency and consistency through standardized output and automated analysis.

Finally, the adoption of automated pathology workflows brings forth a range of ethical challenges, including model bias, transparency of algorithmic decisions, responsibility attribution, and patient privacy protection. Future work will systematically address these critical issues by incorporating fairness evaluation, interpretability safeguards, and data encryption techniques into both model design and deployment processes. These efforts aim to ensure compliance and ethical integrity, supporting the sustainable application of the model in clinical practice.
